# The Long and the Short of It: A Comparison of the Effectiveness of Parent–Child Care (PC–CARE) and Parent–Child Interaction Therapy (PCIT)

**DOI:** 10.1007/s10578-021-01257-9

**Published:** 2021-09-29

**Authors:** Susan G. Timmer, Brandi Hawk, Maria Usacheva, Lindsay Armendariz, Deanna K. Boys, Anthony J. Urquiza

**Affiliations:** Department of Pediatrics, CAARE Diagnostic and Treatment Center, University of California at Davis, Children’s Hospital, 3671 Business Dr., Sacramento, CA 95820 USA

**Keywords:** Brief parenting intervention, Child behavior problems, Treatment outcomes, Treatment fidelity, Treatment comparison

## Abstract

Research shows that parenting interventions struggle with keeping clients in treatment. The purpose of this study was to compare attrition and rates of improvement in caregiver-child dyads participating in either Parent–Child Care (PC–CARE), a brief, 7-session parenting intervention or Parent–Child Interaction Therapy (PCIT) over a 7–week period. Participants were 204 caregiver-child dyads referred to either PC-CARE (N = 69) or PCIT (N = 135) between 2016 and 2019. Children were aged 2–7 years, referred for treatment by county Behavioral Health Services, and Medicaid funded. Findings showed that PC–CARE participants were 2.5 times more likely than PCIT participants to complete 7 sessions, all other things being equal, and showed significantly greater rates of improvement during this timeframe in reported child behavior problems and parenting stress. In conclusion, compared with PCIT, PC–CARE showed greater retention and rate of improvement in child and parent outcomes over a comparable time period.

## Introduction

For many young children referred for mental health services, parenting interventions are often recommended, as these treatments have been found to be highly effective, especially for those with externalizing behavioral problems, such as aggression, defiance, and poor impulse control [1]. However, in studies of parenting interventions, researchers report attrition rates from 50 to 70% [2, 3]. While recent policy advocates the use of evidence-based mental health treatments (e.g., Families First Prevention Services Act– H.R. 5456, FFPSA) and efforts to implement these interventions for children have increased [4], the challenge of engaging and keeping families engaged in treatment remains [5]. One strategy for reaching the largest number of families while maximizing the effectiveness of parenting programs is to develop briefer forms of interventions that can be provided in a variety of settings [6].

## Designing a Brief Parenting Intervention: PC–CARE

Parent–Child Care (PC–CARE) is a brief (1 pre-treatment plus 6 weekly sessions) dyadic parenting intervention for children aged 1–10 years. We developed the protocol using research on effective strategies in parenting interventions [1, 7], coaching [8], and implementation in community mental health settings [9]. We wanted to develop a brief intervention that addressed problems with engagement (i.e., client attrition/retention) reported in research on parenting interventions in community settings [2, 3]. We incorporated strategies for engaging caregiver and child in treatment, such as facilitating positive perceptions and realistic expectations, making assessments meaningful [10], and strengthening the client-centered focus by assessing problems weekly [11]. We also asked them how much they thought the skills we were teaching them would work for them (on a 5-point Likert scale), acknowledging their agency in the decision to use or not use the information we provided and breaking down defensive reactions at being told what to do. Allowing them to give voice to their attitudes and beliefs in this way is consistent with motivational interviewing styles of engagement in treatment [12]. We planned to use a coaching modality with the parent and child together, which has been found to increase the effectiveness of parenting interventions [8]. Finally, the intervention needed to be feasible for use in a community mental health setting. Preliminary results of an open trial were promising, showing that 94% of families completed treatment and reported significant improvements in child behavior problems with large effect sizes (*η*^2^ > 0.17 [23]) and 50% of caregivers reporting child behavior improvements of at least one standard deviation [13]. Improvements in parenting skills, and parenting stress were also statistically significant and with large effect sizes [13]. However, the question of how PC-CARE compares to evidence-based parenting interventions requiring more time to complete remains. To address this goal, this study will compare attrition and treatment gains over a similar timeframe in families referred to PC-CARE with outcomes in families referred to a well-established dyadic treatment, Parent–Child Interaction Therapy [14] (PCIT).

## A Side-by-Side Comparison of PC–CARE and PCIT

### Underlying Theoretical Principles

PCIT and PC–CARE are both based on attachment theories that emphasize the role of caregiver warmth and sensitivity in child adaptive functioning [15] common to many positive parenting approaches [16], behavioral theories that address the power of caregivers’ attention as a social reinforcement of children’s behavior [17], social learning theories that point out the power of children’s observation and imitation of their caregivers in establishing behavioral repertoires [18], and theories of operant conditioning, advocating the importance of consistent consequences and rewarding positive and desired behavior [19].

### Intervention Components

PCIT is a parenting–oriented treatment comprised of two phases, each lasting 7–10 weeks: Child Directed Interaction (CDI), in which therapists focus on parents’ acquisition of positive parenting skills, and Parent Directed Interaction (PDI), in which therapists focus on parents’ ability to give effective commands and timeouts for child noncompliance. PCIT begins with an assessment then a didactic session, in which the therapist teaches specific positive parenting skills to the parent. Subsequent coaching sessions focus solely on improving parents’ positive verbal expression to their children (i.e., increasing parents’ praise, descriptions of their children’s behavior, and reflecting children’s speech) until they reach predetermined goal criteria (i.e., give 10 praises, 10 reflective statements, and 10 descriptions of child’s behavior in a 5-min behavioral observation). Only when caregivers have established a firm foundation of positive verbal expression, do they learn to give effective commands and timeouts as a consequence for their child’s noncompliance with commands.

Unlike PCIT, PC–CARE seeks to maximize skills taught to caregivers while minimizing their time commitment by teaching and coaching caregivers’ use of positive verbalizations alongside behavioral management strategies for six treatment sessions, beginning with the first session. PC–CARE offers a range of strategies that caregivers may choose to use depending on their child’s needs and responsiveness, developmental readiness, and external circumstances. Among these are setting rules, giving positive incentives, and following noncompliance with consistent and logical consequences (e.g., enforcing a rule), such as a removal of a privilege. In addition, PC–CARE aims to support emotional regulation through calming (e.g., belly breathing, mindfulness) and coregulation (e.g., breathing together, back rub) skills, redirection, and giving transitional warnings for changing activities. Engagement and motivational strategies are implemented each session to reinforce the sense of the intervention being a “team” effort, describing what will happen in each session and focusing on identifying which skills are working best for the family (see Table 1 for a comparison of the two interventions).

**Table 1:** Differences between PCIT and PC-CARE

PCIT components	PC–CARE components
***Purpose*** Parents use positive parenting skills taught at a criterion level, give effective commands, and children respond to parent skills	***Purpose*** Expose parents and children to positive parenting skills, strategies to manage behavior, show through coaching what works for them
***Duration*** Between 14 and 20 weeks, depending on caregiver achieving goal criteria for skills taught, reduced child behavior problems	***Duration*** 7 weeks
***Structure*** Teaching, practice until skills are mastered Pre-treatment:—Behavioral observation, orientation to treatment CDI (Child Directed Interaction)—Teach positive attention skills, selective attention (1 session); coach positive attention, selective attention weekly until caregiver achieves mastery of positive parenting skills (7–10 sessions) PDI (Parent–Directed Interaction) —Teach effective commands, timeout procedure (2 sessions), coach commands and timeout until caregiver achieves mastery and child behavior problems are in normal range on standardized assessment (7–10 sessions)	***Structure*** Scaffolded curriculum teaching new skills, coaching previously acquired and new skills, engagement & motivation strategies implemented each session Pre-treatment—Behavioral observation, engagement strategies: orientation to treatment, psychoeducation on reasons for child behavioral problems Session 1—Positive attention, transitions, compliance friendly environment Session 2—Emotional regulation & coping, coregulation, selective attention, modeling, redirecting Session 3—Rules, choices, positive incentives Session 4—Effective commands, logical consequences and removal of privileges for noncompliance Session 5—Recovery, re-do Session 6—Putting it all together: review all strategies to manage child behavior problems
***Mechanism*** Weekly assessment Guided Practice— directive coaching, validating skill use, corrective coaching, higher order statements, psychoeducation Homework—5 min of daily parent–child play	***Mechanism*** Weekly assessment Guided Practice—directive coaching, validating skill use, corrective coaching, higher order statements, psychoeducation Homework—5 min of daily parent–child play, practice strategies throughout the day

PCIT has been found to be effective in reducing children’s disruptive behaviors [14] and maintaining treatment gains for up to 6 years [20]. A recent study presented data showing parents reported improvements in their children’s behavior problems even when terminating treatment early, having at least four treatment sessions focused only on increasing positive parent skills in addition to assessment and didactic sessions [21]. It is possible that the gains parents report from focusing on acquiring positive parenting skills (i.e., CDI) in the first 7 sessions are equivalent to participating in PC–CARE, where they learn behavior management strategies alongside positive parenting skills.

### Other Characteristics

Although both PCIT and PC–CARE aim to improve parent–child relationship and decrease child difficult behaviors and caregiver stress, these two interventions have been developed for somewhat different populations and with different purposes. PCIT was developed for children with severe conduct and emotional regulation problems, including opposition, defiance, aggression, and other externalizing behaviors, while PC–CARE was developed for children with moderate to mild problemss.

## Purpose of the Current Project

The purpose of this project was to compare the retention rates and selected outcomes of two interventions, PC–CARE and PCIT, over a 7-week period. Seven weeks is the time needed to complete PC–CARE and is also the approximate time needed to for participants to complete the first phase of PCIT, that focuses largely on positive parenting skills. Because PC–CARE is faster paced and actively incorporates engagement strategies, we hypothesized that PC–CARE would show better client retention rates within the 7-week timeframe for children referred to either PC–CARE or PCIT between 2016 and 2019. Because PC–CARE incorporates more behavior management strategies than PCIT does in its first phase of treatment, we hypothesized that PC–CARE would show improvement more quickly than PCIT when comparing the two interventions at similar dosage levels (i.e., mid-PCIT vs. post-PC–CARE).

## Methods

### Participant Referral Sources and Funding for Services

All children were referred to a University-connected community mental health agency between 2016 and 2019 to address behavioral problems. Most of the children (61%) were self-referred (i.e., caregiver initiated) often with a social worker’s assistance. Among other referral sources, 16% were referred for treatment by CPS social workers, 11% by primary care physicians, 4% by schools, and 8% by other agencies or therapists. All children were referred for services through the county Department of Behavioral Health and had subsidized insurance (i.e., medicaid), which paid for mental health services. Therapists solicited informed consent from caregivers and verbal assent from children 6 years of age and older to share ownership of their clinical data for research purposes. The University IRB approved the study, consent forms and the use of these data for future research purposes.

### Participants

Children were included in this study if after receiving a thorough assessment and meeting medical necessity per state Medicaid standards, they were referred to PCIT or PC-CARE between January 2016 and November 2019. Additionally, children were included only if funded by the Early and Periodic Screening, Diagnostic and Treatment (EPSDT) benefit, which requires Medicaid enrollment and ensured that participants were similarly resourced. In all, 109 children were referred to PC–CARE and 137 children were referred to PCIT for treatment of their disruptive or difficult-to-manage behaviors. However, the age range for PC–CARE participants (1–10 years) was wider than it was for PCIT participants (2 –8 years). So, for the purposes of this study, we excluded children younger than 2 years and older than 7.9 years to have comparable age groups for participants of both interventions. The result was a total of 69 PC–CARE and 135 PCIT participants.

Children in the sample ranged in age from 2.10 to 7.83 years (mean = 4.95, SD 1.6) and 63% were male. Approximately 36% of the children were Caucasian, 27% were African American, 29% were Latinx, and 8% were other races/ethnicities. Similarly, approximately 40% of the caregivers were Caucasian, 24% were African American, 26% were Latinx, and 10% were other ethnicities. The majority of children participated in treatment with their biological parents (73%); 13% participated with relative caregivers, 14.6% with nonrelative caregivers. All families were low-income; two-thirds of the families in the sample (66.5%) reported earning $20-$25,000 a year or less. Children’s diagnoses fell into four major categories (numbers do not add to 100% because children could have more than one diagnosis or a diagnosis that fell into more than one category): adjustment disorders (26%), disruptive behavior and conduct disorders (52%), emotional disorders (e.g., anxiety, depression; 29%), and trauma-related disorders (e.g., PTSD; 8%).

#### Reasons for Referral to PC–CARE

Because the clinic provided both PCIT and PC–CARE, two parenting interventions with similar mechanisms of change, we needed to be purposeful in deciding which clients should be referred for PC–CARE vs. a longer treatment like PCIT. When clients were eligible for both PCIT and PC–CARE, clients may have been referred to PC–CARE ultimately for several reasons: (1) the caregiver would not or could not commit to participate in a 12–20 week intervention; (2) the child was receiving individual therapy (e.g., Trauma-Focused Cognitive Behavioral Therapy) and needed a supplemental parenting intervention to move forward in therapy; (3) children with caregivers who had mental health problems or recent substance abuse problems were perceived as needing more time to acquire needed parenting skills and so were referred to PCIT over PC–CARE; (4) caregivers were given a choice of the two interventions and chose PC-CARE over PCIT.

### Treatment Process

#### Description of PC–CARE Intervention Structure/Curriculum and Mode of Delivery

At the start of PC–CARE, the pre-treatment session is dedicated to assessment and orientation to treatment. Providers begin treatment by checking in with the child and caregiver and discussing the outcomes of any assessments that have been administered, then they conduct a behavioral observation of the parent and child playing together, during which the provider codes the parent’s verbalizations (e.g., positive verbalizations (i.e., PRIDE skills), questions, commands). Following this, the provider briefly discusses the behavioral observation, then explains how environmental and/or physiological factors (e.g., exposure to violence or traumatizing event, developmental delay, chronic illness) can contribute to the development of negative behavior repertoires. Next, the provider reviews with caregivers and children what the goals of PC–CARE are and what will happen each week. This pre-treatment session is followed by six intervention sessions. Participants that “dropped out” of treatment had at least one treatment session before terminating; participants that “never started” treatment completed the pre-treatment assessment but terminated before treatment sessions began.

Each 1-h intervention session includes a check-in (7 min), teaching parenting skills (10 min), observational assessment (4 min), coaching the caregiver to use the skills taught (20 min), and wrap-up (10 min). In addition to the hour-long treatment session, parents are asked to spend 5 min per day in play with their child and to use the new skills at home throughout the day. Parents document their playtime and skill use on a “Daily Care” sheet.

#### Description of PCIT Intervention Structure/Curriculum and Mode of Delivery

At the start of PCIT, the first session is spent conducting a 15-min parent–child observational assessment, during which the therapist codes parents’ verbalizations, debriefing, and orienting the parent to treatment. Providers typically review the parent’s goals for treatment and how PCIT can help them. Following this session, parents come in without the child for a session in which the therapist teaches the parent about positive parenting skills (e.g., PRIDE skills)—what they are, how and why they should use them, and using selective attention to reduce difficult behaviors. After this session, parents are coached to use these skills until they reach protocol-determined goal criteria of using a specific number of positive parenting skills (10 praises, 10 reflections, 10 behavioral descriptions, no more than 3 questions, commands or negative talk) during a 5-min behavioral observation. Once they achieve the goal criteria, the caregivers attend another teaching session without their children, in which they receive training on how to give effective commands and timeouts when needed. Caregivers are coached to use the commands and timeouts until the child complies consistently with commands. Participants that “dropped out” of treatment had at least one coaching session before terminating; participants that “never started” treatment completed the pre-treatment assessment but terminated before coaching sessions began.

The 1-h coaching sessions include check-in (10 min), observational assessment (5 min), coaching (30 min), wrap-up and check-out (15 min). In addition to the treatment sessions, parents are asked to spend 5 min a day playing with their child.

### Setting

Services were provided by licensed and license–eligible mental health providers working at a university hospital-associated community mental health center that primarily served children with subsidized health care. The interventions were either provided in the home setting (14%) or in the clinic (86%). Clinic rooms furnished with a two-way mirror and audio/visual equipment were used to deliver both PCIT and PC–CARE in the clinic. PCIT and PC–CARE providers taught skills (i.e., didactics) in the therapy room but coached caregivers from an observation room behind the two-way mirror, using a single-frequency receiver and earpiece so that the children could not hear the coach and their focus would be on their parent. Children whose families were not able to access services at the clinic (e.g., no transportation, several young children in the household) could request equivalent in-home services. All components of in-home treatment were the same, except that the therapist coached the caregiver while seated in the same room (e.g., behind or next to the caregiver) rather than from behind a mirror but staying out of the play.

### Measures

#### Eyberg Child Behavior Inventory (ECBI)

The ECBI [22] is a 36–item scale that measures disruptive behavior problems exhibited by children aged 2–16 years. Caregivers are asked to report the frequency of certain behaviors, such as “Acts defiant when told to do something” or “Sasses adults” (Intensity scale) and whether these behaviors are considered to be problems (Problem Scale). The ECBI has been standardized on several populations [22]. Test–retest correlations across a 3-week time span on the ECBI Intensity and Problem scales were 0.86 and 0.88 respectively. The published cut-off scores for child problem behaviors are an intensity score of greater than 131 or a problem score of greater than 16.

#### Parenting Stress Index, 4th Ed.– Short Form (PSI4–SF)

The PSI4–SF [23] is a 36–item questionnaire that generates a Total Parenting Stress score, based on subscale scores measuring perceived stress from the demands of the parenting role (Parental Distress; e.g., “I feel trapped by my responsibilities as a parent.”), from problems in the parent–child relationship (Parent–Child Dysfunctional Relationship; e.g., “My child rarely does things for me that make me feel good.”), and because of the child’s difficult behaviors or temperament (Difficult Child; e.g., “My child’s behavior is more of a problem than I expected.”). Caregivers respond to 36 questions with a 5-point Likert scale (1 = Strongly Agree to 5 = Strongly Disagree). Raw scores are then transformed into percentile scores. Analyses of internal consistency revealed alpha coefficients of reliability of α = 0.90 for the Parental Distress scale, α = 0.89 for the Parent–Child Dysfunctional Relationship scale, and α = 0.88 for the Difficult Child scale.

#### Family Demographic Characteristics

The “Brief Family Life Questionnaire” (BFLQ) [24] asks parents to provide information about children’s family demographic characteristics, such as ethnicity, household income and composition, and how long the child has been in the caregiver’s custody.

#### Diagnostic Category

Therapists used the DSM–5 as a guide for diagnosing the child clients, recording the corresponding ICD–10 codes in client plans and county files. In order to reduce the large number of different diagnoses into a smaller number of categories, we coded diagnoses into four different categories: (1) adjustment disorders; (2) conduct and disruptive behavior disorders; (3) emotion/mood disorders (e.g., anxiety, depression); and (4) traumatic stress-related disorders. If a diagnosis fell into more than one category, they were coded in both categories. For instance, a child diagnosed with Adjustment disorder with anxiety would be coded as having an adjustment related disorder and an emotion/mood related disorder.

### Fidelity of Treatment Provision

To ensure that PCIT and PC–CARE participants received the services per respective evidence-based protocols, 37% of PCIT cases (N = 48) and 36% of PC–CARE cases (N = 25) were reviewed for fidelity.

#### PCIT Fidelity

Fidelity for PCIT cases was evaluated based on the following criteria as applicable: whether 15-min behavioral observations were conducted at pre-, mid-, and post-treatment, and 5-min observations were conducted at the start of each session; whether teaching sessions were conducted at the beginning of each phase of treatment (CDI and PDI); whether positive parenting (PRIDE) skills were coached during CDI and effective commands with time out as a backup for noncompliance were coached in PDI. Adherence to these processes was evaluated by reviewing casefiles and progress notes. If reviewers found evidence of weekly coding or coaching the elements meant to be covered in that phase of treatment (e.g., presence of completed coding sheets and/or case notes mentioning coding outcomes, caregiver response to coaching PRIDE skills, or direct commands and timeout), they noted their presence on a fidelity worksheet.

We found that 98% of cases showed evidence of having conducted the pre-treatment behavioral observation, 100% the mid-treatment behavioral observation, 83% of those completing treatment had a post-treatment behavioral observation, and 98% conducted weekly behavioral observations. Based on evidence from case notes, all participants received a CDI didactic session, CDI before PDI content, and were coached to use PRIDE skills in CDI. Eighty percent of mid-treatment sessions occurred between sessions 7 and 11; 76% of caregivers reached a preset criterion level of competence in PRIDE skills before moving on to the second phase of treatment. Approximately 98% of those participating in the second phase of treatment had documentation of a PDI teach session, and 95% had documentation related to having coached effective commands and compliance in PDI. These statistics suggest that PCIT was provided with adequate fidelity to protocol.

#### PC–CARE Fidelity

PC–CARE cases’ fidelity was evaluated by reviewing a randomly selected recording of one of the participant’s sessions. Using an in-house fidelity worksheet, reviewers noted how much time the therapist spent giving the didactic information and coaching, and whether the information and coaching were session specific.

Reviews of session videos from the 25 different PC-CARE clients showed that all required skills were taught and coached in 70% of sessions reviewed. The average amount of time spent teaching in each session was 8.5 min (SD 3.0 min), ranging between 3 and 15 min (target time—10 min); 76% of session didactics were 10 min long or less. The average time spent coaching was 15.6 min (SD 5.9 min), ranging between 4 and 24 min of coaching; 76% of sessions reviewed had 10 min or more of coaching, 56% had 15 min or more of coaching. These statistics suggest that PC-CARE was provided with adequate fidelity to protocol.

### Analysis Strategy

We will explore the significance of differences between PCIT and PC–CARE participants using Chi-square tests and analyses of variance (ANOVAs). Significant differences between sample populations will be covaried in subsequent analyses to test for differences in retention/engagement and select performance outcome measures.

To probe for differences in treatment engagement of PC–CARE and PCIT participants, we examined marginal differences and conducted a binomial logistic regression, which estimates the odds of terminating treatment early and whether it varies by treatment group, controlling for group differences. Because PCIT takes longer to complete than PC-CARE, we also compared children completing at least 7 sessions of PCIT with the number of children completing the 7-session PC–CARE intervention, examining marginal differences and conducting a binomial logistic regression, to see whether PC–CARE and PCIT are equally effective in keeping clients engaged during the first 7 weeks of the intervention, all other things being equal.

In evaluating the relative effectiveness of the two interventions, we conducted a repeated measures analysis of covariance (MANCOVA), with assessment point as the repeated measure and intervention type as the between–subjects measure, covarying characteristics that differentiated the two intervention populations. We used an alpha of 0.05 in all analyses. Analyses of the ECBI and PSI4–SF were conducted separately to maximize sample size. An average sample size of 55–60 in our analyses of treatment effects was sufficient to detect medium effect sizes with a power of 0.80. In addition to the observed power of treatment effects, we presented *η*^2^ (eta-squared) for analyses of variance and a *φ* (phi) for Chi-square tests, statistics that indicate the proportion of variance accounted for by membership in the designated group (i.e., the between–subjects factor). Eta-squared is roughly the square of *F*, the statistic measuring effect size in analyses of variance. A small effect size for an analysis of variance is *F* = 0.10 (*η*^2^ = 0.01), a medium effect size is *F* = 0.25 (*η*^2^ = 0.06), and a large effect size is *F* = 0.40 (*η*^2^ = 0.16). In a 2 × 2 cross-tabulation, *φ* is equal to the effect size indicator *w*, in which a small effect size is *w* = 0.10, medium effect size is *w* = 0.30, and large effect size is *w* = 0.50 [25].

All dyads described above were eligible for inclusion in the analyses described below. However, sample sizes varied because of missing data on different outcome measures.

## Results

### Descriptive Differences

Results of the Chi-square analyses of demographic characteristics showed that PC–CARE participants were more likely to be female [*χ*^2^ (1, N = 204) = 4.6, *p* = 0.03] and less likely to be accompanied by biological parents in treatment than were PCIT participants [*χ*^2^ (2, N = 204) = 8.82, *p* = 0.01]. In terms of diagnoses, PC–CARE participants were more likely than PCIT participants to have been diagnosed with adjustment related disorders, [*χ*^2^ (1, N = 204) = 5.70, *p* = 0.02]. Results of a two-way between-subjects ANOVA showed that children referred to PC–CARE in this sample were on average younger than those referred to PCIT, although the range in children’s ages were matched for each sample to account for the more restricted age range of PCIT participants [F(1, 204) = 5.0, *p* = 0.03]. Children and caregivers in the two interventions did not differ in their race, ethnicity, or referral source (see Table 1).

As far as the level of behavioral problems at pre-treatment, both the intensity and numbers of behavior problems reported by caregivers on the ECBI were significantly higher for children participating in PCIT compared to PC–CARE, (intensity: F(1, 182) = 9.1 *p* = 0.003; number: F(1, 182) = 13.8 *p* < 0.001). A post-hoc analysis showed that at pre-treatment, 63% of PCIT participants had scores that fell within the clinical range on the ECBI Intensity scale (i.e., T-scores > 60), compared to 43.5% of children participating in PC–CARE [*χ*^2^ (1, N = 204) = 7.05, *p* = 0.008, φ = − 0.19].

### Differences in Client Retention

An examination of group differences in the percentage of children completing, terminating early, and never starting treatment (see Table 2) showed that 84.1% of children completed PC–CARE compared with 45.9% completing PCIT [*χ*^2^ (2, N = 204) = 28.7, *p* < 0.001]; and 65.1% of children completed at least 7 sessions of PCIT [*χ*^2^ (2, N = 204) = 7.99, *p* = 0.005].

**Table 2:** Descriptive differences between dyads participating in PCIT and PC–CARE

Session	PCIT (N = 135)	PC–CARE (N = 69)	Effects
Child’s age	5.11 (1.5)	4.59 (1.7)	F(1, 204) = 5.0, *p* = ..03, *η*^2^ = 0.02, OP = 0.60
Child’s sex (% male)	68.9%	53.6%	*χ*^2^ (1, N = 204) = 4.6, *p* = 0.03, φ = – 0.15
Child’s ethnicity			*χ*^2^ (3, N = 202) = 2.95, *p* = 0.40, φ = 0.12
% African American	28.0	26.1	
% Caucasian	32.8	43.5	
% Latinx	27.1	26.1	
% Other race/ethnicity	8.8	4.3	
Diagnosis			
% Conduct problems	47.4	52.2	*χ*^2^ (1, N = 204) = 0.42, *p* = 0.52, φ = 0.05
% Adjustment	20.7	36.2	*χ*^2^ (1, N = 204) = 5.70, *p* = 0.02, φ = 0.17
% Mood/emotion	25.9	36.2	*χ*^2^ (1, N = 204) = 2.34, *p* = 0.13, φ = 0.11
% Traumatic stress	8.3	8.8	*χ*^2^ (1, N = 204) = 0.02, *p* = .90, φ = 0.01
Relationship of caregiver			*χ*^2^ (2, N = 204) = 8.82, *p* = 0.01, φ = 0.21
% Biological parent	79.3	60.9	
% Relative caregiver	8.1	20.3	
% Non–relative cgiver	12.6	18.8	
Caregiver’s ethnicity			*χ*^2^ (3, N = 202) = 2.27, *p* = 0.52, φ = 0.11
% African American	24.8	23.2	
% Caucasian	36.8	44.9	
% Latinx	27.1	26.1	
% Other race/ethnicity	11.3	5.8	
Referral type			*χ*^2^ (3, N = 202) = 6.18, *p* = 0.103, φ = 0.17
% CPS referral	12.7	21.7	
% Self-referral	59.7	63.8	
% Physician	14.2	5.8	
% School/other	13.4	8.7	
Severity of behavior problems (ECBI)	(N = 120)	(N = 68)	
Mean intensity	64.7 (10.7)	58.2 (9.6)	F(1, 182) = 9.1, *p* = 0.003, *η*^2^ = .0.05, OP = 0.85
Mean # of problems	67.2 (10.1)	59.9 (10.6)	F(1, 182) = 13.8, *p* < 0.001, *η*^2^ = 0.07, OP = 0.96

To test for potential differences in the odds of dropping out of treatment prematurely, we conducted a stepwise binomial logistic regression of dropping or never starting treatment on type of intervention (i.e., PCIT vs. PC–CARE participants). At step 1, based on the descriptive group differences, we controlled for children’s age, gender, whether they were diagnosed with an adjustment disorder, whether they were participating in treatment with a biological parent, and whether the severity of their behavior problems was reported to be in the clinical range pre-treatment. We found that children referred to PCIT were more than twice as likely to leave treatment within the first 7 weeks than children referred to PC–CARE [Exp (B) = 2.5, p = 0.02]. No other indicators significantly predicted early treatment termination (see Table 3).

**Table 3:** Differences between dyads participating in PCIT and PC–CARE

	PCIT (N = 135)	PC–CARE (N = 69)	Effects
Treatment progress			
% Complete treatment	45.9%	84.1%	*χ*^2^ (2, N = 204) = 28.7, *p* < 0.001, φ = 0.38
% Drop early	41.5	8.7	
% Never start	12.6	7.2	
% Complete 7 weeks	65.2	84.1	*χ*^2^ (2, N = 204) = 7.99, *p* = 0.005, φ = − 0.20

At step 2, we added an interaction term between intervention type and the severity of child behavior problems (normal vs. clinical range), to determine whether higher levels of behavior problems increased the likelihood of dropping out of either intervention. We found no significant interaction effects between clinical behavioral problems and dropping out of either PCIT or PC-CARE within the first 6 coaching/treatment sessions (change in *χ*^2^ = 0.66, df = 1, 204, *p* = 0.42).

### Differences in Rate of Improvement

We conducted repeated measures analysis of covariance (RMANCOVA) to investigate the differences between performance outcomes of PCIT vs. PC–CARE participants after approximately 7 weeks of treatment. We first explored group differences between parental ratings of children’s behavior and self-reported parenting stress levels at similar dosages of treatment: mid-treatment of PCIT (approximately 7–11 weeks of treatment), focus on positive parenting and non-directive methods of behavioral control, and post-PC–CARE completion (see Table 4).

**Table 4:** Results of a binomial logistic regression predicting the likelihood of early treatment termination within the first 7 sessions (N = 204)

Independent variables	Early treatment termination within first 7 sessions		
	B (SE)	Exp (B)	Effects
PCIT (1) vs. PC–CARE (0)	0.93 (0.40)	2.50	*p* = 0.02
Child’s age	0.20 (0.11)	1.23	*p* = 0.06
Sex of child (M/F)	0.06 (0.34)	1.09	*p* = 0.85
Caregiver biological parent (1)	0.67 (0.43)	1.94	*p* = 0.12
Adjustment–related disorder (1)	– 0.10 (0.38)	0.82	*p* = 0.80
ECBI Intensity (normal/clinical)	– 0.50 (0.34)	0.61	*p* = 0.15
Model statistics	− 2LL = 225.98 χ^2^ = 17.59, df = 6, 204, *p* = 0.007 Cox & Snell R^2^ = 0.08		

 As shown in Table 5, the analyses that compared PCIT participants’ mid-treatment ratings (which typically occurred between sessions 7 and 11) with PC–CARE participants’ post-treatment ratings (which occurred at session 7) revealed significantly greater improvements of the frequency and numbers of behavior problems, and total parenting stress in children participating in PC–CARE compared with children participating in the first phase of PCIT. Results also show significant main effects for assessment point differences in the frequency of problem behaviors and parenting stress [ECBI intensity: F(1, 121) = 18.70, *p* < 0.001, *η*^2^ = 0.13, OP = 0.99]; suggesting that participants in both interventions showed significant gains in the time period examined on these outcome measures. Analyses also showed significant main effects for intervention type for all three measures, showing that children in PCIT were reported as having more severe behavior problems and that caregivers had higher parenting stress than PC–CARE participants across both assessment points. As a post hoc analysis, we reran the RMANCOVAs including an intervention type (PCIT vs. PC–CARE) by behavior problem severity (normal vs. clinical range) interaction term to determine whether children with more severe behavior problems performed differently in PCIT vs. PC–CARE. None of the interaction terms significantly predicted symptom change (see Table 5).

**Table 5:** Means, standard deviations, and results of repeated measures analyses of covariance by treatment type

Dependent variables	PCIT outcomes		PC–CARE outcomes		Effects
	PRE–treatment	MID treatment*	PRE–treatment	POST–treatment**	
ECBI Intensity Scale (N^pcit^ = 69, N^pccare^ = 57)	63.8 (10.7)	62.1 (9.0)	57.8 (9.8)	49.3 (11.0)	A × I: F(1, 121) = 25.01, *p* < 0.001, *η*^2^ = 0.17, OP = 10.0
					A: F(1, 121) = 0.03, *p* = 0.85, *η*^2^ = 0.00, OP = .05
					I: F(1, 121) = 18.70, *p* < 0.001, *η*^2^ = 0.13, OP = 0.99
					B: F(1, 121) = 123.3, *p* < 0.001, *η*^2^ = 0.51, OP = 1.0
ECBI Problem Scale (N^pcit^ = 68, N^pccare^ = 56)	65.9 (10.6)	68.4 (12.5)	59.3 (10.7)	53.9 (12.8)	A × I: F(1, 119) = 12.6, *p* = 0.001, *η*^2^ = 0.10, OP = 0.94
					A: F(1, 119) = .60, * p* = .44, *η*^2^ = .0.005, OP = 0.12
					I: F(1, 119) = 16.17, *p* < 0.001, *η*^2^ = 0.12, OP = 0.98
					B: F(1, 119) = 63.7, *p* < 0.001, *η*^2^ = 0.35, OP = 1.0
PSI4– SF, Total stress (N^pcit^ = 65, N^pccare^ = 56)	69.2 (23.9)	69.2 (21.9)	59.41 (26.5)	44.34 (29.8)	A × I: F(1, 116) = 17.2, *p* < 0.001, *η*^2^ = 0.13, OP = 0.99
					A: F(1, 116) = 2.88, *p* = 0.09, *η*^2^ = 0.02, OP = 0.39
					I: F(1, 116) = 7.04, *p* = 0.009, *η*^2^ = 0.06, OP = 0.75
					B: F(1, 116) = 31.5, p < 0.001, *η*^2^ = 0.21, OP = 1.0

*Repeated measure* assessment point (pre, approximately session 7); *Covariates* caregiver is biological parent (no/yes), diagnosis is adjustment disorder (no/yes), child behavior problems (normal/clinical)

+ A = Assessment point; I = Intervention type; B = Behavioral risk; A × I = Assessment point by intervention type interaction

*Occurs between session 7–11, depending on speed of client progress

**Occurs session 7

## Discussion

The purpose of this study was to compare treatment retention and client outcomes in two dyadic parenting interventions in order to evaluate the costs and benefits of using a brief parenting intervention, PC–CARE, over the longer intervention, PCIT. PC–CARE was developed as a way to provide parents with basic parenting and behavior management skills before they terminated therapy, which we gauged at 7 sessions based on previous research findings [26]. We also believed that PC–CARE might be sufficient for families reporting less severe behavior problems, benefitting families by requiring less time commitment while also reducing the costs to the healthcare system. For these reasons, we hypothesized that PC–CARE might outperform PCIT when comparing outcomes along a similar timeline (i.e., seven weeks).

Results of analyses confirmed our hypotheses that PC–CARE outperformed PCIT in retaining caregivers and children in treatment during the first seven weeks of treatment (PCIT = 65.2% vs PC–CARE = 84.1% completing 7 weeks of treatment). We argue that participants in PC–CARE were more likely to complete treatment than PCIT participants were to complete a similar number of sessions because we incorporated evidence-based principles of engagement [10, 11] into the fabric of treatment: participants had clear information about what would happen each session, caregiver and child had a voice in the treatment process, and we presented parenting skills as a selection of strategies from which they could choose the most effective for them.

Results of analyses also confirmed that PC–CARE participants reported significantly greater improvements in the number and intensity of child behavior problems, and in total parenting stress in seven weeks, from pre- to post-treatment, than PCIT participants reported in a similar timeframe, from pre- to mid-treatment. These findings suggest that in this population of children and caregivers, only working to increase parents’ positive parenting skills predicts significant improvements in children’s behavior problems but adding to the curriculum strategies to manage difficult behavior, calming and co-regulation, including the child, and a focus on what works best for the family may increase gains.

Preliminary analyses revealed that PCIT and PC–CARE participants differed significantly in the level of symptoms endorsed pre-treatment; caregivers participating in PCIT endorsed higher levels of child behavior problems and parenting stress pre-treatment than PC–CARE participants. This difference may be a result of therapists’ increased likelihood of referring children with fewer behavior problems to PC-CARE. However, to control for the possibility that the initial severity of children’s behavior problems accounted for differences in treatment outcomes, we covaried whether children had clinically significant levels of behavior problems, which accounted for a significant amount of variation in comparisons of the interventions’ change in these analyses. It did not, however, account for observed differences between PCIT vs. PC–CARE participants’ performance after approximately 7 sessions. To ensure that these PC–CARE participants’ greater improvement was a result of protocol differences rather than population differences, we ran analyses to check whether participants with clinical levels of initial behavior problems improved similarly in PC–CARE and PCIT. Analyses consistently showed that PC–CARE participants reported significantly more improvement in behavior when compared with PCIT participants measured in a comparable timeframe, whether those behaviors were initially in the clinical or normal range.

### Limitations of the Current Study

One limitation of this study is that children were not randomly assigned to treatment. This is a quasi-experimental study comparing these two interventions: children referred to our clinic for treatment of disruptive behavior problems during the same timeframe, all low income, through our county’s Department of Behavioral Health and funded with Medicaid dollars, and were either recommended for PCIT or PC–CARE by the therapist conducting the intake assessment. As can be seen by mean levels of behavior problems in children initiating PC–CARE vs. PCIT, children with less severe behavior problems and less stressed caregivers tended to be assigned to PC–CARE, although there was considerable overlap in the types of clients assigned to each intervention. While this structure allowed us to compare the performance and engagement of dyads participating in these two interventions, there were differences in the two populations of participants that needed to be covaried. Other unmeasured differences might also have existed.

Another limitation of the study was that fidelity was evaluated differently for PC-CARE and PCIT participants. While we had video recordings of most PC-CARE clients’ sessions, we depended on therapists’ case notes to obtain information about the content of PCIT sessions. Having video of PC-CARE sessions allowed us to be fairly granular and strict with assessments of its fidelity. With only case notes for PCIT clients, we needed to focus on the structure of the intervention and basic content to avoid biased assessments of fidelity.

Last, because children in this study were typical community mental health clients, it may be more difficult to generalize to the population of families with private insurance or paying out of pocket for services.

## Conclusion

Results of analyses suggested that PC–CARE is a parenting intervention that participants were significantly more likely to complete compared with those referred to a longer intervention, staying involved long enough to learn skills for managing their children’s difficult behavior as well as positive parenting skills, and also reporting significant treatment gains. The findings reported here represent a step in constructing the evidence base for this brief intervention.

## Summary

Research shows that parenting interventions struggle with keeping clients in treatment, suggesting there may be some benefit to investigating the relative effectiveness of briefer interventions. The purpose of this study was to compare attrition and rates of improvement in caregiver-child dyads participating in either PC–CARE, a brief, 7-session parenting intervention or Parent–Child Interaction Therapy (PCIT) over a 7–week period. Participants were 204 caregiver-child dyads referred to either PC-CARE (N = 69) or PCIT (N = 135) between 2016 and 2019. Children were aged 2–7 years, referred for treatment by county Behavioral Health Services, and Medicaid funded. Findings showed that participants in the brief intervention, PC–CARE, were 2.5 times more likely than participants in the longer intervention, PCIT, to complete 7 sessions, all other things being equal, and showed significantly greater rates of improvement during this timeframe in reported child behavior problems and parenting stress. In sum, PC–CARE participants were found to be significantly more likely to complete treatment compared with those referred to a longer intervention, staying involved long enough to learn skills for managing their children’s difficult behavior as well as positive parenting skills, and reporting significant treatment gains. The findings reported here support the use of brief parenting interventions to treat moderate child behavior problems.
